# Core concepts in statistics and research methods. Part 5: systematic review and meta-analysis

**DOI:** 10.1016/j.bjae.2025.10.002

**Published:** 2025-11-05

**Authors:** B. Doleman, J.G. Hardman

**Affiliations:** 1 University of Nottingham, Nottingham, UK; 2 Nottingham University Hospitals NHS Trust, Nottingham, UK

**Keywords:** meta-analysis, publication bias, systematic review

**Learning objectives**

By reading this article, you should be able to:

• Recall what a systematic review, with or without a meta-analysis, is and when these analyses should be performed.
• Appreciate how the use of certainty of evidence influences the position of meta-analyses in the hierarchy of evidence.
• Recognise the biases and errors that can occur in meta-analyses.
• Practically appraise meta-analyses for use in clinical decision-making.

**Key points**

• Systematic reviews and meta-analyses have traditionally been placed at the peak of the hierarchy of evidence.
• Issues with bias and error mean that high-certainty evidence from meta-analyses is rare.
• The quality of evidence provided by meta-analyses are summarised within the GRADE framework, which includes risk of bias in included studies, publication bias, indirectness, imprecision and heterogeneity.

A systematic review is defined as a synthesis of individual studies with a clearly defined research question that uses explicit methods to identify and appraise all of the available evidence and present the results. There are two methods of presenting the results. One approach is to present a narrative synthesis, which involves a descriptive discussion. The second approach is to do a meta-analysis, which involves statistical pooling of the estimates from the individual studies.^1^

Meta-analyses have many advantages over individual studies, including increased statistical power, identifying treatment effects in different groups of patients and the ability to investigate what makes treatments more or less effective.

Many of the statistical concepts discussed in this article rely on knowledge of study types and statistical principles, such as standard error (se), *p*-values and confidence intervals (CIs), which have been discussed in previous articles in this series.^2^^,^^3^ A clinical scenario is presented to illustrate the issues discussed (Box 1).^4^

Box 1 **Clinical example**

A consultant anaesthetist is concerned about the development of chronic postsurgical pain (CPSP) after 6 months in patients undergoing mastectomy. She wishes to determine whether i.v. lidocaine can reduce the risk of this complication. She investigates the evidence and finds a recently published meta-analysis on the subject.^4^ The results of the meta-analysis suggest there is no reduction in CPSP with an odds ratio of 0.48 (95% confidence interval, 0.19–1.21). There was modest statistical heterogeneity (*I*^2^ = 32%). However, she would like to know how much certainty she can place in these findings, given she recalls that meta-analyses of randomised controlled trials (RCTs) sit at the top of the hierarchy of evidence.

Alt-text: Box 1

## Hierarchy of evidence

**Fig 1:**
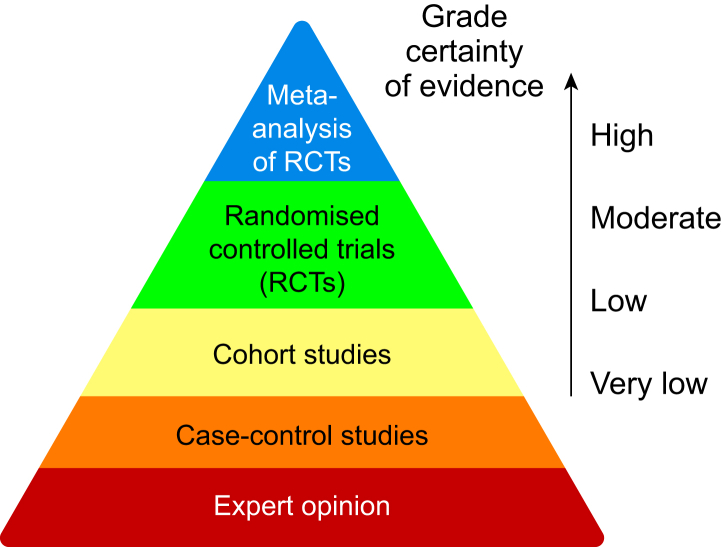
Traditional hierarchy of evidence, with meta-analyses of randomised controlled trials at the summit. The arrow on the right demonstrates that if certainty of evidence is reduced, then the position on this hierarchy is modified. GRADE, Grading of Recommendations, Assessment, Development and Evaluations.

In the traditional hierarchy of evidence, meta-analyses of RCTs sit at the pinnacle (Fig. 1). However, there are issues with almost all meta-analyses that mean this position is not fully justified and that estimates from meta-analyses may be biased.^5^ One issue concerns the types of studies that can be included in a meta-analyses, which are often observational. Meta-analyses of observational studies are common when RCTs are deemed unethical or impractical, for example when quantifying the frequency of serious adverse events. A useful method of quantifying the quality of evidence from systematic reviews and meta-analyses is to use a tool such as the Grading of Recommendations, Assessment, Development and Evaluations (GRADE) framework (Table 1).^6^

**Table 1:** Grading of Recommendations, Assessment, Development and Evaluations (GRADE) framework with examples of one-level and two-level downgrading. Assessments are subjective, so the examples here may be rated differently by different assessors.

Domain	Description	One level downgrade	Two level downgrade
Risk of bias	Assessment of the risk of bias in the included studies. These include selection bias, performance bias, attrition bias and selective outcome reporting (Table 2).	Many studies are rated as some concerns and one study high risk of bias.	Almost all studies rated as high risk of bias and no studies low risk of bias.
Inconsistency	Describes the degree of statistical heterogeneity in the estimates.	Evidence of moderate statistical heterogeneity (*I*^2^ = 51%).	Evidence of considerable heterogeneity (*I*^2^ = 99%).
Indirectness	Similar concept to external validity. Describes evidence derived from a meta-analysis that is in a different population to the one the results will be applied to.	Meta-analysis of studies using non-opioid analgesics in breast surgery where results will be applied to laparoscopic cholecystectomy. Effects may still apply to some degree (reduce pain).	Evidence from anaesthetic technique used in aortic valve replacement via sternotomy applied to transcatheter aortic valve implantation.
Imprecision	How close effect estimates are likely to be to the population value. Assessed using confidence interval width or using trial sequential analysis information size.	Risk ratio 1 (95% confidence interval, 0.5–2)	Risk ratio 1 (95% confidence interval, 0.1–10)
Publication bias	Asymmetric funnel plot or statistically significant quantitative test. Suggests possible preferential publication of positive studies.	Four studies on one side of the bottom right portion of the plot with no corresponding negative studies on the other side.	Fifteen studies on one side of the bottom right portion of the plot with no corresponding negative studies on the other side.

Meta-analyses of observational studies are lower in the evidence hierarchy than meta-analyses of RCTs, because of inherent biases and confounding with the former. Bias and confounding in observational studies are discussed in a previous article in the series.^3^ However, because they provide a summary of the best available evidence, meta-analysis of observational studies make an important contribution to the medical literature. Even though RCTs are theoretically less prone to bias and confounding than observational evidence, a study has suggested that estimates from observational meta-analyses give similar results to those derived from RCTs.^7^ Furthermore, meta-analyses of small RCTs have poor concordance with the results from large, well-designed RCTs.^8^ This finding may be partly because research demonstrating high-certainty evidence from meta-analyses of RCTs in anaesthesia is rare.^9^

## Research question, literature search and study selection

A well-defined research question is an essential component of a systematic review. The research question is best structured using a population, intervention, control, outcomes and statistical analysis (PICOS) question. A thorough search strategy is important in order to capture all the available evidence to answer the research question. The main electronic databases for a systematic review include the Medical Literature, Analysis and Retrieval System Online (known as MEDLINE), Embase and the Cochrane Central Register of Controlled Trials (known as CENTRAL). Developing a search strategy can benefit from the expertise of a clinical librarian and is aided by using sensitive search filters from the Cochrane handbook of systematic reviews.^10^

The first domain of GRADE we will discuss is publication bias, detailed below. Publication bias describes a phenomenon where studies showing statistically significant results are more likely to be published (and are published faster) than studies showing non-significant results. Publication bias creates a distortion in the medical literature, where the accumulation of published papers gives an overly optimistic evidence base for an intervention. Publication bias is common within the anaesthesia literature.^11^ It is, therefore, essential that authors of systematic reviews conduct a thorough search for unpublished studies. Sources of unpublished studies include clinical trial databases (e.g. Clinicaltrials.gov), grey literature and conference proceedings. Authors of unpublished studies should be contacted to identify whether any study data exist, which can then be incorporated into the review. However, response rates from the authors of unpublished studies are low and incorporating data that has not undergone peer review may introduce bias into the review.

Once searches are complete, study selection should be conducted by two review authors, aiming to reduce biases in the inclusion process and reduce the number of errors. For example, one author may have a biased view of a particular intervention and may be more likely to include or exclude studies. To aid in study selection, explicit inclusion and exclusion criteria should be mapped to the PICOS question.

Registering the review before starting is an essential step and a common requirement of journals for subsequent publication. Registration can be with the Open Science Framework or the International Prospective Register of Systematic Reviews (known as PROSPERO).^12^ Registration helps reduce duplication, increase transparency and prevent the selective reporting of outcomes. For example, review authors may be tempted to add additional outcomes if they identify beneficial effects during the data extraction process (selective outcome reporting bias). Reporting guidelines (e.g. the Preferred Reporting Items for Systematic Reviews and Meta-Analyses, known as PRISMA) help ensure all methodological features are reported and are associated with improved review quality.^5^

## Quality assessment

An appraisal of the included evidence is of fundamental importance, because a meta-analysis is only as good as the quality of the included studies. For RCTs, the most commonly used and up-to-date method of quality assessment is the Cochrane Risk of Bias Tool (Version 2).^13^ The Cochrane Risk of Bias Tool assigns ‘low risk’, ‘some concerns’ and ‘high risk’ to various aspects of trial conduct that can cause systematic bias. The domains and examples of ‘low risk’ and ‘high risk’ of bias are provided in Table 2.

**Table 2:** Cochrane Risk of Bias Version 2 examples of low risk and high risk of bias using scenarios in acute pain randomised controlled trials.

Domain	Description	Low-risk example	High-risk example
Bias arising from the randomisation process	Uses information on how the randomisation list was created and how allocation to groups was concealed from investigators. Also uses baseline characteristics to determine if the randomised groups are similar.	• Randomisation list was created using a computer and therefore can be regarded as truly random. • This list was produced and kept in pharmacy. Investigators had no access, so could not know which group the next potential participant would be allocated to. • Baseline characteristics of the groups are very similar.	• Participants were allocated to groups based on what day they had surgery. This is not random. • As patients were allocated based on the day of the week, investigators knew which group the next potential study participant would be allocated to. Therefore, they try to not recruit people to the intervention group who they think will have more pain, so the intervention appears more beneficial (*selection bias*).
Bias due to deviations from intended interventions	Identifies whether participants and personnel were aware of the assigned intervention, and whether deviations from intended interventions were related to the effect being estimated.	• I.V. lidocaine was given along with a saline placebo. These were prepared by pharmacy and were identical so participants and anaesthetists did not know which was which.	• Oral pregabalin was given 1 h before surgery with no placebo. Participants knew if they were receiving the intervention, which improved pain via the *placebo effect*. Anaesthetists knew groups, so gave more analgesia to the intervention group.
Bias due to missing outcome data	Assesses the amount, reasons and handling of missing outcome data and whether these could bias results.	• All participants were analysed so there was no missing data	• 50% of participants were excluded from the placebo group because of extreme pain. This would make the intervention appear less effective (*attrition bias*).
Bias in measurement of the outcome	Considers whether outcome assessors were blinded, if measurement methods were appropriate and whether assessment could have been influenced by knowledge of the intervention.	• Pain was measured using a visual analogue scale, which has been validated, and outcome assessors were blinded.	• Outcome assessors knew that participants received pregabalin, as postoperative doses were given in front of them and no placebo was used. Outcome assessors tried to lead patients on pain scores to make the intervention appear more effective.
Bias in selection of the reported result	Examines whether reported analyses match the prespecified analysis plan and whether multiple analyses were possible, with selective reporting of favourable results.	• Investigators recorded eight outcomes on Clinicaltrials.gov before the start of the study and reported all of these as specified.	• Investigators only preregistered pain measurement but included multiple opioid adverse events and multiple different analyses of pain until they found a significant difference.

In Table 2, the presence of a single instance of ‘high risk’ of bias in one domain would lead to an overall high risk of bias for the study and the potential downgrading of evidence from GRADE (Table 1). From Table 2, we can see that if studies are labelled as RCTs but do not use a true random procedure or a placebo, then the evidence is at risk of similar biases and confounding as observational evidence and, therefore, should be treated as such in terms of the hierarchy of evidence (Fig. 1).

Considering our clinical scenario, when examining the meta-analysis of chronic postsurgical pain (CPSP) at 6 months after surgery, the studies included were at high risk of bias because of deviations from the intended interventions and bias in the outcome measurement. Therefore, the evidence could reasonably be downgraded owing to concerns over risk of bias in the included studies.

## Data extraction and analysis

As with the decision-making on including and excluding studies, two review authors should extract the data to improve data accuracy.^14^ As mentioned previously, one of the main advantages of meta-analysis is the increase in power that can be achieved by combining the effect estimates from multiple individual trials. Combining studies together increases the effective sample size and therefore the precision of the estimates (i.e. narrower CI).^2^ A common method of combining studies is a technique called generic inverse variance.

### Generic inverse variance

Generic inverse variance assigns a weight to a study, so that when all studies are analysed together, larger studies have more influence on the overall results. The term variance refers to the square of the se (i.e. se^2^). A detailed discussion of the se can be found in a previous article in the series.^2^ A worked example using generic inverse variance for two hypothetical studies is provided in Box 2.

Box 2 **A simple worked example of generic inverse variance using a fixed-effect model**

To combine two studies using generic inverse variance, we require the effect estimates and standard errors (ses) for the included studies. Consider two hypothetical randomised trials (A and B) comparing the effectiveness of a new beta-blocker (reductolol) for treating hypertension. In both studies, reductolol resulted in a larger decrease in arterial systolic blood pressure than the control beta-blocker. Study A has a larger sample size than study B and so has a smaller se. The results are:

**Table:** 

	Effect estimate	se
Study A	3.0 mmHg	1.0
Study B	6.1 mmHg	1.4

***Weighting for each study***

The weighting for each study is calculated as:

$$w=\frac{1}{{SE}^{2}}$$

Where ${SE}^{2}$ is the square of the standard error, which is the sampling variance. So, the weightings for studies A and B are approximately 1 and 0.5, respectively.

***Pooled estimate of the standard error***

The pooled estimate for the standard error for the two studies is calculated as:

$$SE=\sqrt{\frac{1}{w_{A}+w_{B}}}=\sqrt{\frac{1}{\;1+0.5}}=\sqrt{\frac{1}{1.5}}≃0.82$$

***Pooled estimate of the effect***

The pooled estimate of the effect for the two studies is the weighted average of the effect estimates (EAs) for the two studies:

$$\text{Pooled}\;\text{estimate}=\frac{{EA}_{A}\times w_{A}+{EA}_{B}\times w_{B}}{w_{A}+w_{B}}=\frac{3\times 1+6.1\times 0.5}{1+0.5}≃4\;\text{mmHg}$$

Notice that the pooled estimate of effect (4 mmHg) is closer to Study A (3 mmHg), which is the larger of the two studies. Notice also that the pooled se (0.82) is smaller than the ses of the individual studies, reflective of the larger sample size when the two studies are combined.

This example is illustrative only. In real meta-analyses, the calculations are more involved, as typically data from several studies are combined.

Alt-text: Box 2

When using generic inverse variance, we are assuming that all studies are estimating a single common effect (fixed effect). Where we assume the effects in the included studies are from a distribution of different effects (random effects), then equation (2) (see section on heterogeneity below) should be substituted into the denominator of the first equation in Box 2.

### Forest plots

**Fig 2:**
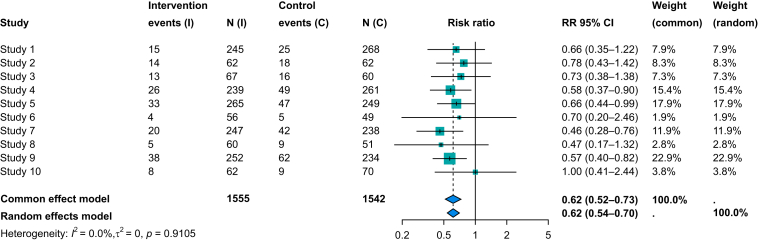
Forest plot of simulated randomised controlled trials involving a binary outcome. From columns left to right: name of the studies is on left of the plot, the next four columns contain data from the number of events and sample size in the intervention and control group, respectively. Effect estimates and 95% confidence intervals (CIs) with study weights are on the right of the plot. Larger studies (smaller standard errors) will generally have narrower CIs and larger boxes for weights (compare Study 6 and Study 9). The blue diamond shows the pooled effect estimate (centre of the diamond) and the width of the diamond represents the 95% CI for the meta-analysis for a random- and fixed (common)-effect model. RR, relative risk.

Results from meta-analyses are most commonly presented using a forest plot (Fig. 2). The left side of the plot contains the authors’ names and year of publication. Each study estimate is represented by a square and the corresponding 95% CI shown as a horizontal line. Larger studies generally have smaller ses and therefore narrower CIs; this effect can be seen in Figure 2, where Study 9 (*n* = 486) has a narrower CI than Study 6 (*n* = 105). The estimated pooled effect is shown as a diamond, where the centre of the diamond is the effect estimate and the width of the diamond is the pooled 95% CI. The forest plot provides a visual demonstration of how pooling estimates from multiple studies decreases the width of the CI and therefore increases precision.^2^

Although power is increased with meta-analysis, there may still be wide CIs around the pooled estimates, indicating imprecision. In our clinical scenario (Box 1), the width of the 95% CI ranged from an approximately 80% reduction to a 20% increase (Box 1). The wide CI means we are very uncertain about where the population effect lies. On GRADE assessment, evidence could be downgraded because of imprecision.

## Dichotomous and continuous outcomes

Outcomes are broadly categorised as binary (e.g. the occurrence of postoperative vomiting) or continuous (e.g. a visual analogue score for pain). For binary outcomes, an odds ratio (OR), risk ratio (RR; relative risk) or risk difference can be used to quantify effect estimates. An OR is the ratio of events versus non-events in both groups. The OR has favourable mathematical properties, although it can be misinterpreted by clinicians, who often interpret ORs as RRs.^15^ The RR is subtlety different, as it is defined as the ratio of risks between groups. Risk can be calculated within each group by dividing the number of events in each group by the group size. The RR is usually what clinicians think of as risk, so it would usually be preferred to ORs. When events are rare, ORs and RRs are similar, but they can be markedly different when events are common. In our example forest plot (Fig. 2), the RR is 0.62, which means a 38% reduction in the risk of the event with the intervention. Details on how the OR and RRs are calculated can be found in a previous article in this series.^2^

For continuous outcomes, the main methods of displaying effect estimates are the mean difference (MD) and the standardised mean difference (SMD). The MD is simply the difference in the means between the groups. The SMD is the MD divided by the pooled standard deviation:

(1) $$SMD=\frac{MD}{{SD}_{\text{pooled}}}$$

The SMD allows the meta-analysis of the same outcome measured on different scales, for example visual analogue scales (0–100 mm) and numeric rating scales (0–10). However, because SMDs lose the original units, it becomes more difficult to judge the clinical significance of a pooled effect. For example, a reduction of 9.9 mm on a visual analogue scale is regarded as a clinically significant reduction in acute pain.^16^ Conversion of an observed MD in the visual analogue scale to an SMD would not allow the 9.9 mm threshold to be applied. Although cut-offs do exist for SMD estimates, these are arbitrary selections rather than those derived from specific evidence.

Returning to our clinical scenario (Box 1), the result for CPSP at 6 months was expressed as an OR. As the anaesthetist would like to know the effect estimate and 95% CI in terms of risk (e.g. a RR or risk difference), the effect estimate was difficult for her to interpret.

## Fixed- and random-effects models

Authors need to decide what model to use for the meta-analysis. The two types of models are fixed (or common) and random effects. A fixed-effect model assumes there is only one underlying treatment effect to estimate. For example, if two studies were analysed, which involved identical interventions in identical groups of patients (e.g. same hospital), and were conducted in an identical manner, then the assumption of a fixed-effect model would be satisfied. However, the assumptions for fixed effects are very rarely satisfied and, therefore, random-effects models should be used for most meta-analyses. Random-effects models assume treatment effects arise from a distribution of effects, for example, a normal distribution. Random-effects models introduce additional variance (variability) to the results, as shown in Equation 2:

(2) $$v_{i}^{T}=v_{i}+\tau^{2}$$

In equation (2), $v_{i}^{T}$ is the total variance of study i, $v_{i}$ is the within-study variance for study i and $\tau^{2}$ (‘tau squared’) is the between-study variance. The within-study variance is the square of the se for an individual study (i.e. $v_{i}={\text{SE}}_{i}^{2}$) . If the effects observed across studies are very different, then total variance is greatly increased. Incorporating both forms of variance leads to wider CIs around the effect estimates for a random-effects model than a fixed-effects model.

A common misconception is that the choice of model (fixed *vs* random effects) should be based on some measure of statistical heterogeneity (discussed below). However, the decision should be based on the assumptions of the model rather than a specific result from a statistic. From our clinical scenario, a random-effects model was used, which was appropriate given there were differences in study groups, lidocaine dosing regimens and methodologies across the included studies.

## Heterogeneity

### Clinical heterogeneity

Clinical heterogeneity is an important aspect for authors of meta-analyses to consider. Clinical heterogeneity describes differences between the included studies, which can include differences between patients included, interventions and study conduct mentioned previously. If too much clinical heterogeneity is present, then a meta-analysis is not appropriate. Excess clinical heterogeneity can lead to the criticism that a meta-analysis is comparing ‘apples and oranges’.

### Statistical heterogeneity

Statistical heterogeneity is a quantitative measure of how effects in each study differ by more than would be expected by chance. Two commonly reported measures of statistical heterogeneity are the between-study variance ($\tau^{2}$), introduced earlier, and the *I*^2^ statistic. We can think of τ as a standard deviation of effect estimates, so this gives a measure of how ‘spread out’ the effects are across studies. We can see from Figure 2 that the 95% CIs overlap, indicating the effects are similar across the studies and, therefore, the value of τ^2^ is zero.

The *I*^2^ statistic quantifies the percentage of the total variability that results from differences between studies rather than sampling variability (i.e. within-study variance). For instance, an *I*^2^ value of 95% suggests that almost all the total variability is a result of differences in the effects between the studies. Notice in Figure 2 that the *I*^2^ statistic is zero, indicating the variance is entirely caused by within-study variance.

From our clinical scenario, there was modest statistical heterogeneity (*I*^2^ = 32%, Box 1), which would result in downgrading the evidence as per GRADE (Table 1). After removal of a study that was clinically heterogeneous (a regional technique used), the statistical heterogeneity disappeared (*I*^2^ = 0%). Excluding the heterogeneous study would avoid downgrading the evidence.

### Prediction intervals

Prediction intervals are an alternative method of quantifying heterogeneity. Prediction intervals provide an interval estimate within which we would expect a new study effect to fall. In the presence of high statistical heterogeneity, the prediction interval is wide, indicating we are more uncertain where an effect for a new trial would be observed.

### Subgroup analysis and meta-regression

Although substantial statistical heterogeneity can be viewed as a problem in a meta-analysis (using a random-effects model in the presence of high statistical heterogeneity results in wider CIs and larger *p-*values), heterogeneity has some benefits. For instance, we may obtain important clinical information from observed differences in effects across studies. For example, we can use subgroup analysis to see whether the addition of a postoperative infusion of ketamine is more effective that an intraoperative infusion alone. However, subgroup analyses are often underpowered.

**Fig 3:**
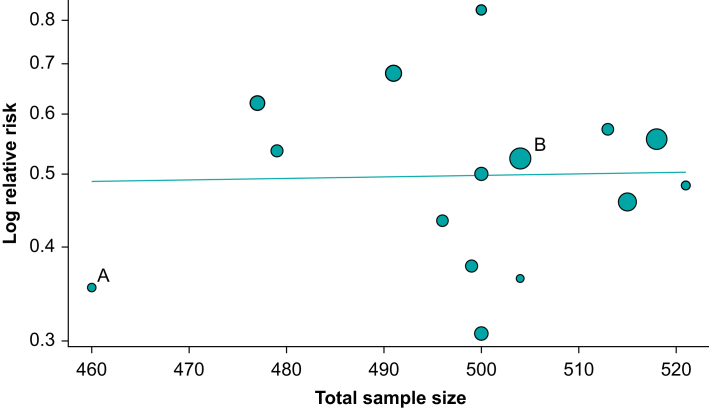
Bubble plot showing the effect estimate (vertical axis) on a logarithm scale predicted by total sample size (horizontal axis). The horizontal line is the (linear) regression line. The slope is close to 0, which demonstrates that total sample size does not predict the effects in the studies. Point A indicates a smaller study with smaller weight and point B indicates a larger study with larger weight. However, this relationship is influenced by other factors than purely sample size, such as the number of events in each group.

A more elegant technique than subgroup analysis is to use a form of linear regression called meta-regression. Meta-regression uses study-level predictor variables (e.g. the dose of a drug used in the study) to predict the outcome (effect estimate in the study) (equation [3]). Meta-regression is almost identical to standard linear regression except that studies are used instead of participants, and each study needs to be weighted (see Box 2), so larger studies have a greater effect on the effect estimate. The effect of different study weights can be seen in Figure 3, where a small study (A) is represented by a small bubble and a larger study (B) by a large bubble. We also need to include between-study error to a random-effects meta-regression, given as:

(3) $$\theta_{i}=\beta_{0}+\beta_{1}\times {\text{Dose}}_{i1}+u_{i}+\varepsilon_{i}$$

In equation (3), $\theta_{i}$ is the effect in study i, $\beta_{0}$ is the intercept coefficient, $\beta_{1}$ is the effect (slope) coefficient, ${\text{Dose}}_{i1}$ is the dose in study i, $u_{i}$ is the between-study error (i.e. heterogeneity) and $\varepsilon_{i}$ is the within-study error (i.e. sampling variability).

For illustrative purposes, in Figure 3, we try to predict study outcome by total sample size. The line of best fit is horizontal, suggesting that total sample size is not predictive of the study outcome (i.e. the slope coefficient is close to zero). Meta-regression does have issues, namely ecological bias and the same issues that affect observational research, such as confounding.^3^ Ecological bias describes associations that can occur at an aggregate level (e.g. the mean age of participants in a study) but not at an individual level (e.g. participant age). Confounding occurs because studies are not randomised to particular covariates. Multivariable meta-regression can be used by adding additional confounding variables to equation (3), as in observational research designs.^3^

## Publication bias

As described previously, publication bias involves the preferential publication of studies reporting statistically significant results.^11^ Publication bias means that when authors of systematic reviews attempt to locate studies from electronic databases, the sample of studies may be biased towards those demonstrating benefit with the intervention. Various plots and statistical tests can be used to try to identify publication bias in meta-analyses. The most common technique is to use a funnel plot.

### Funnel plots

**Fig 4:**
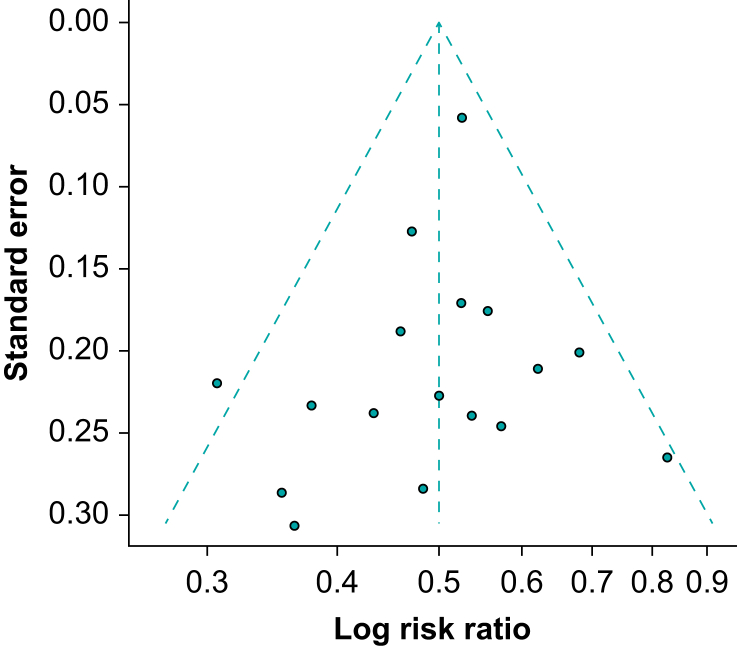
Funnel plot showing the effect estimate on the horizontal axis (log risk ratio) and the standard error (se) on a reverse scale on the vertical axis. Studies are plotted as circles. Larger studies (low se) are towards the top of the plot, with estimates lying close to that of the meta-analysis. Small studies (higher se) are towards to the bottom of the plot and are more dispersed as described in the text. The plot is symmetrical with a roughly even spread of studies within the funnel, and therefore no evidence of small study effects (lower risk of publication bias).

A funnel plot (Fig. 4) displays the effect estimate on the horizontal axis and a measure of sampling variance on the vertical axis, usually the se on a reverse scale. Larger studies (with smaller se) aggregate towards the peak of the plot and should be closer to the true population value, whereas smaller studies (larger se) will usually have effects that are dispersed further from the overall effect, creating a funnel shape. The funnel shape is also related to the central limit theorem, which is discussed in the first article in the series.^2^

Some authors argue that visual inspection of funnel plots can be inaccurate,^5^ so statistical tests can be used to identify funnel plot asymmetry. One well-known example is Egger’s linear regression test, which can help quantify any asymmetry by reporting a *p-*value.

## Types of meta-analysis

Thus far, we have generally discussed meta-analyses comparing a single intervention with a control group or another intervention, which are called conventional or pairwise meta-analyses. However, what happens when there are multiple potential interventions for a condition and we wish to identify which is the most effective? Returning to our clinical scenario, our consultant anaesthetist has been discussing the evidence for i.v. lidocaine with colleagues, who have raised concerns regarding potential local anaesthetic toxicity. So, she begins to consider other potential interventions, although wonders how to identify the best treatment. Such clinical questions on relative efficacy may be answered by using a network meta-analysis.^17^

### Network meta-analysis

Network meta-analysis allows comparison of multiple interventions for a clinical condition by using two types of estimates—direct and indirect—and combines them to form one estimate called a mixed estimate. Advantages include an increase in power, the ability to compare treatments not directly compared in trials and the ability to rank treatments. A major disadvantage is the main assumption of transitivity. Transitivity means that effect modifiers are equally distributed between treatment comparisons, which may be an unrealistic assumption.^17^ For this reason, the relative merits of two treatments are more accurately assessed with a large RCT comparing the two treatments directly.

Direct estimates from network meta-analysis are identical to the estimates from a conventional (pairwise) meta-analysis, which is where interventions are directly compared within a study to yield a single estimate. An indirect estimate involves calculating effects from interventions that have not been directly compared within a trial. For example, if paracetamol reduces acute pain scores by 10 mm (on a 100-mm visual analogue scale) compared with placebo, and ibuprofen reduces pain by 20 mm compared with placebo, we can calculate that ibuprofen reduces pain by 10 mm when compared with paracetamol. Indirect estimates mean we can compare the effects of paracetamol and ibuprofen, even if no RCT has directly compared these two treatments. With this in mind, our consultant anaesthetist identifies a network meta-analysis evaluating non-opioids for CPSP, which identifies i.v. lidocaine as the most effective treatment.^18^

### Trial sequential meta-analysis

**Fig 5:**
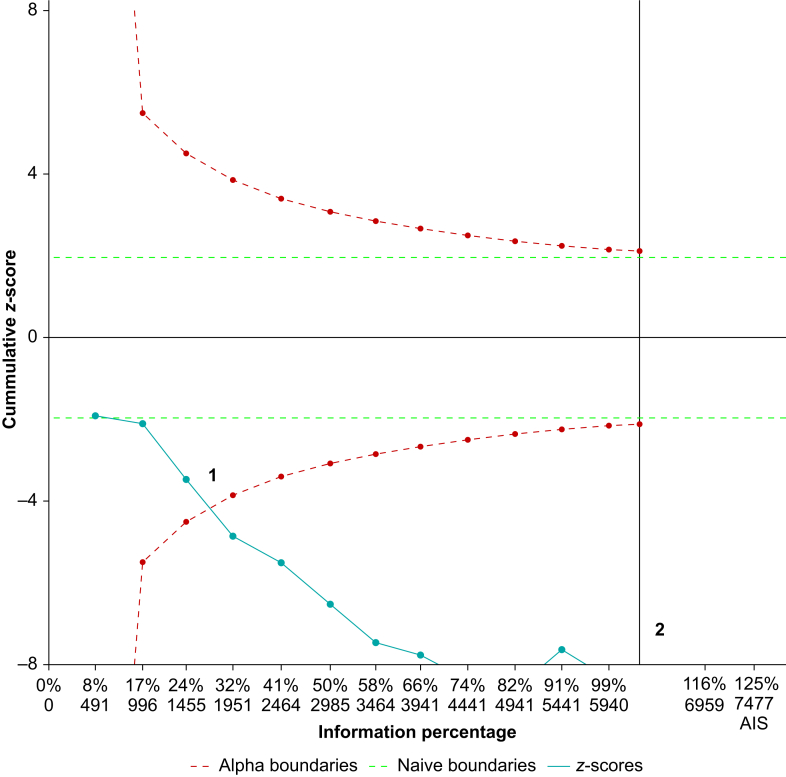
Trial sequential analysis plot. Cumulative *z*-score is on the vertical axis and number of participants in the included trials on the horizontal axis. The plot demonstrates that with a lower number of participants, a larger degree of statistical significance (larger *z*-score) is required to demonstrate benefit (red dashed line). Point 1 demonstrates the effect on meta-analysis (teal line) crossing this boundary and thus showing a benefit adjusted for multiple comparisons (reduces the probability of a type I error). Point 2 demonstrates the required sample size of 6,261 (information size) required to reduce the probability of a type II error. If this point is reached without a statistically significant effect, a type II error is unlikely.

In primary research studies where data are analysed using null hypothesis statistical testing, it is possible to make errors.^2^ Rejection of the null hypothesis when it is true is a type I error (false positive) and failure to reject the null hypothesis when it is false is a type II error (false negative). These errors can still occur in meta-analyses because the publication of each new RCT offers an opportunity for another meta-analysis and multiple testing increases the probability of a type I error. Furthermore, despite one of the main advantages being increased statistical power, meta-analyses can still be underpowered, resulting in an increased probability of a type II error. Trial sequential meta-analysis is a technique designed to reduce statistical errors. Type I errors are minimised by requiring smaller *p-*values (larger *z*-scores) early in evidence accrual. We can see this in Figure 5, where the effects cross the adjusted boundary for statistical significance (point 1 on the plot). Type II errors are reduced by calculating the sample size required to achieve a desired power, called the information size (point 2 on Fig. 5). Using trial sequential analysis, Imberger and colleagues^19^ demonstrated that in a sample of anaesthetic meta-analyses, only 12% had a power of >80% and only 32% kept the type I error rates <5%.

### Individual patient data meta-analysis

All the examples of meta-analyses discussed so far use studies as the unit of analysis. However, using the original data from all included RCTs, it is possible to perform an individual patient data meta-analysis. Individual patient data meta-analysis still requires that such analyses preserve information about where the data came from, because data within each RCT will be correlated. Dealing with correlation requires advanced statistical methods.

Advantages of individual patient data meta-analysis include the ability to investigate subgroups more accurately, handle missing data more appropriately, adjust for confounding variables and analyse data using consistent methods (Table 3). Disadvantages include difficulties obtaining the original patient-level data, negotiating collaboration recognition and the increases in resources required.^20^

**Table 3:** Description of the different types of meta-analyses including a summary of the approach and key features.

Type of meta-analysis	Description	Key features
Pairwise meta-analysis	Compares a single intervention with a control group or another intervention using direct estimates from studies. Can use RCTs or observational evidence.	• Single direct comparison and estimate • Can include multiple outcomes
Network meta-analysis	Allows comparison of multiple interventions by combining direct and indirect estimates to produce mixed estimates.	• Enables ranking of treatments • Can compare interventions even without direct head-to-head trials • Additional assumption required (*transitivity*)
Trial sequential analysis	Adjusts meta-analysis boundaries to reduce type I errors and calculates required information size.	• Reduces type I errors • Determines if sufficient evidence has accumulated (reduces type II errors)
Individual patient data meta-analysis	Uses original patient-level data from trials rather than summary data.	• Allows detailed subgroup analysis • Better handling of missing data • Resource intensive • Can perform regression on individual participants • Modelling of non-linear relationships • Performance of time-to-event analyses
Bayesian meta-analysis	Uses Bayesian statistical methods to pool data.	• Can incorporate previous information • Results expressed as credible intervals that allow probability statements • Flexible for complex models, especially for network meta-analyses

### Bayesian meta-analysis

Meta-analyses can be performed using Bayesian methods. Bayesian inference yields credible intervals as opposed to CIs. The essentials of Bayesian inference have been detailed in a previous article in this journal.^21^ Advantages of Bayesian meta-analyses include incorporating previous information (which could be observational evidence in the case of an RCT-based meta-analysis), easier interpretation of imprecision using credible intervals and greater flexibility, especially for network meta-analyses. A summary of the different types of meta-analysis can be found in Table 3.

## Using GRADE to determine certainty of evidence

Returning to our clinical scenario (Box 1), the point estimate suggests a 50% reduction in the odds of CPSP. However, the CI is very wide, which results in downgrading of precision by two levels. The analysis included two trials with high risk of bias and one trial with some concerns, so this domain was downgraded by one level. The risk of publication bias in the review was low. Evidence was not downgraded for indirectness, as the review was conducted in patients undergoing breast surgery, which is relevant to her target group. Therefore, the overall certainty of evidence was very low. Given her concerns over local anaesthetic toxicity and the imprecision of the estimates, she decided there was insufficient evidence to use lidocaine in her patients.

It should be noted that GRADE assessments are subjective. Therefore, two clinicians may reach different decisions on the certainty of evidence from the same meta-analysis.

## Artificial intelligence

**Fig 6:**
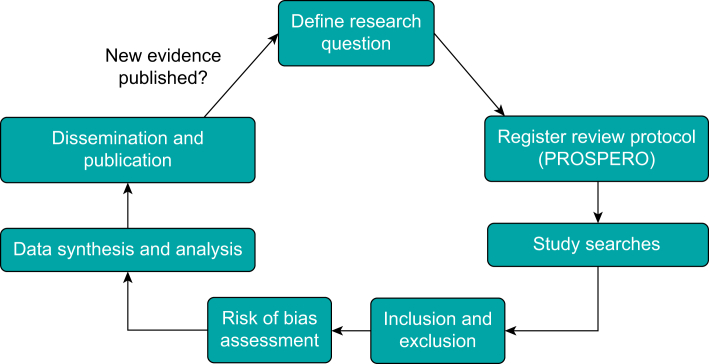
The process of undertaking and completing a meta-analysis. This is presented as a cycle, as further meta-analyses should be conducted as new evidence emerges.

Artificial intelligence has the potential to revolutionise the future of systematic reviews and meta-analyses. Many aspects of the review process (Fig. 6) involve decisions that can be conducted effectively by large language models. Steps suited to automation include searching for studies, determining inclusion and exclusion of trials, assessing the risk of bias and data analysis. Artificial intelligence has the potential to fully automate the review process resulting in increases in efficiency, improved accuracy and allowing real-time updating of meta-analysis results as new studies are published.

## Conclusions

Meta-analyses of RCTs have traditionally held the position at the top of the evidence hierarchy. However, the results from meta-analyses need to be taken in the context of the certainty of evidence; high-certainty evidence from meta-analyses is rare in anaesthesia. Methodological problems with the conduct of meta-analyses should be considered using the GRADE framework, and clinical decisions made accordingly.

## MCQs

The associated MCQs (to support CME/CPD activity) are accessible at www.bjaed.org/cme/home for subscribers to *BJA* *Education*.

## Declaration of interests

JGH is the associate Editor-In-Chief of the *British Journal of Anaesthesia*. BD is an editorial fellow for the *British Journal of Anaesthesia*. The contents of this article represent the experience of the authors and a presentation of the reviewed evidence. It does not represent the policy of the *British Journal of Anaesthesia*.
